# Impact of increased mean arterial pressure on skin microcirculatory oxygenation in vasopressor-requiring septic patients: an interventional study

**DOI:** 10.1186/s13613-019-0572-1

**Published:** 2019-08-29

**Authors:** Sigita Kazune, Anastasija Caica, Einars Luksevics, Karina Volceka, Andris Grabovskis

**Affiliations:** 1 Department of Anesthesiology, Hospital of Traumatology and Orthopedics, 22 Duntes Street, Riga, 1013 Latvia; 2 Laboratory of Biophotonics, Institute of Atomic Physics and Spectroscopy, University of Latvia, 3 Jelgavas Street, Riga, 1004 Latvia; 3 Department of Human and Animal Physiology, Faculty of Biology, University of Latvia, 1 Jelgavas Street, Riga, 1004 Latvia; 4 Clinic of Toxicology and Sepsis, Riga East University Hospital, 2 Hipokrata Street, Riga, 1038 Latvia

**Keywords:** Septic shock, Mean arterial pressure, Tissue oxygenation, Noradrenaline

## Abstract

**Background:**

Heterogeneity of microvascular blood flow leading to tissue hypoxia is a common finding in patients with septic shock. It may be related to suboptimal systemic perfusion pressure and lead to organ failure. Mapping of skin microcirculatory oxygen saturation and relative hemoglobin concentration using hyperspectral imaging allows to identify heterogeneity of perfusion and perform targeted measurement of oxygenation. We hypothesized that increasing mean arterial pressure would result in improved oxygenation in areas of the skin with most microvascular blood pooling.

**Methods:**

We included adult patients admitted to the intensive care unit within the previous 24 h with sepsis and receiving a noradrenaline infusion. Skin oxygen saturation was measured using hyperspectral imaging-based method at baseline and after the increase in mean arterial pressure by 20 mm Hg by titration of noradrenaline doses. The primary outcome was an increase in skin oxygen saturation depending upon disease severity.

**Results:**

We studied 30 patients with septic shock. Median skin oxygen saturation changed from 26.0 (24.5–27.0) % at baseline to 30.0 (29.0–31.0) % after increase in mean arterial pressure (*p* = 0.04). After adjustment for baseline saturation, patients with higher SOFA scores achieved higher oxygen saturation after the intervention (*r*^2^ = 0.21; *p* = 0.02). Skin oxygen saturation measured at higher pressure was found to be marginally predictive of mortality (OR: 1.10; 95% CI 1.00–1.23; *p* = 0.053).

**Conclusions:**

Improvement of microcirculatory oxygenation can be achieved with an increase in mean arterial pressure in most patients. Response to study intervention is proportional to disease severity.

## Background

Tissues depend upon microcirculation, a network of vessels of less than 100 μm in diameter, for delivery of oxygenated blood [1]. Failure of microcirculation to match oxygen supply to demand at the cellular level is thought to be the main mechanism leading to organ failure in septic shock [2]. The goal of early resuscitation in sepsis is to optimize blood flow and restore oxygen delivery to the tissues by the use of fluid resuscitation, inotropic agents and vasopressors. However, it has been shown that microcirculatory abnormalities can persist despite the achievement of microcirculatory targets proposed by international guidelines [3]. Suboptimal perfusion pressure might be an important contributor to microcirculatory abnormalities in septic shock. Due to vasodilatation, fluid resuscitation is not sufficient to increase perfusion pressure in most patients. Increasing mean arterial pressure (MAP) with the administration of noradrenaline is the intervention of choice [4].

Noninvasive techniques based on either measurement of microcirculatory flow or tissue oxygenation have been used to characterize microcirculation in the sublingual mucosa, muscle and skin of septic patients. A known feature of microcirculation in sepsis is its heterogeneity, with areas of preserved blood flow coexisting with non-perfused areas [5–7]. Under conditions of heterogeneous circulation, adequate tissue oxygenation cannot be achieved unless blood carrying oxygen is properly distributed in the tissues. Patient resuscitation in the early stages of sepsis might potentially be improved if the presence of hypoxic areas in the tissues is accurately assessed.

In the skin, heterogeneity of perfusion can be seen as mottling. The characteristic discoloration in mottling is created by the pattern of light reflected from the skin surface. It reflects the relative concentration and distribution of chromophores, including oxy- and deoxyhemoglobin contained in the microcirculation. The light absorption properties of these chromophores are known, and measurements of diffuse reflectance spectra can be used to determine their relative concentrations. The hyperspectral camera combines digital photography with measurement of reflected light intensity at multiple spectral bands for each pixel. Hyperspectral imaging (HSI) enables precise mapping of oxy- and deoxyhemoglobin distribution in the dermal layer of the skin. When microcirculation is heterogeneous, hyperspectral images allow to identify poorly oxygenated areas and perform targeted tissue oxygen saturation measurements there [8].

Thooft et al. have shown that increasing MAP by noradrenaline during septic shock improved the perfused vessel density and the microvascular flow index but not tissue oxygenation measured by near-infrared spectroscopy [9]. In contrast, Jhanji et al. showed an improvement in cutaneous microvascular flow and tissue oxygenation but not sublingual microvascular flow with increasing noradrenaline doses [10]. In both of these studies, measurement of tissue oxygenation was not targeted to areas that were hypoxic at baseline, therefore could have benefitted most from an increase in systemic perfusion pressure.

We aimed to assess the effects of increased mean arterial pressure on microcirculatory saturation in hypoxic skin areas in patients with septic shock using hyperspectral imaging. We hypothesized that increasing MAP by 20 mm Hg by an escalation of noradrenaline doses will result in increase in skin oxygen saturation in these areas.

## Methods

After approval by the Scientific Research Ethics Committee of the Institute of Cardiology and Regenerative Medicine (26/23.02.2017), consecutive vasopressor-requiring adult patients with sepsis admitted within the previous 24 h were recruited from a single 16-bed intensive care unit. We defined vasopressor-requiring septic patients as patients with sepsis or septic shock defined according to The Third International Consensus Definitions for Sepsis and Septic Shock [11] who had received at least 20 ml/kg of crystalloid during the first 4 h of resuscitation, and who required noradrenaline to maintain optimal MAP for at least 4 h. Intensive care physicians could individualize MAP according to the perception of clinical need. Written informed consent was given by all patients or their next of kin before inclusion into the study. We excluded patients with acute myocardial ischemia, arrhythmias, pregnancy and extensive wounds or inflammation in the lower thigh and knee area. Subject demographic characteristics, admission diagnosis, clinical and laboratory variables necessary for the calculation of Acute Physiology and Chronic Health Evaluation (APACHE) II [12] and Sequential Organ Failure Assessment (SOFA) [13] were recorded. Mottling score, which describes the extent of the mottled area of the knee and thigh, was determined on a 6-point scale ranging from 0 to 5 [14]. Arterial lactate concentration measured within 6 h of enrollment and volume of fluid resuscitation from admission to enrollment was also recorded. Data were also collected regarding the need for mechanical ventilation and renal replacement therapy. We also studied a group of healthy volunteers recruited from the hospital staff.

### General management of patients

A radial artery and a central venous catheter were in situ in all patients, and noradrenaline was infused through a dedicated lumen. The hemodynamic management of patients was performed by intensive care physicians according to local protocols. All patients received initial fluid resuscitation of 20 ml/kg of crystalloid solution. Additional fluid boluses were given if judged to improve available hemodynamic variables (blood pressure, heart rate, urine output or pulse pressure variability). The decision to start noradrenaline was taken by intensive care physicians if the patient had signs of shock evidenced by fluid unresponsive arterial hypotension (MAP ≤ 65 mm Hg) associated with clinical signs of inadequate peripheral perfusion and rate of infusion individualized to obtain clinical improvement [15].

### Hyperspectral imaging

The study was performed in the intensive care unit at the bedside with windows shaded and ambient light dimmed to limit light interference. The acquisition of images was performed using the hyperspectral camera Nuance EX (Cambridge Research & Inst., UK) which allows acquisition of high-resolution (1392 × 1024 pixel) image sets in the range of 470–820 nm by the step 5 nm. The camera was equipped with a 60-mm f2.8D lens (AF Micro Nikkor, Nikon, Japan), and a coaxial platform with 5x185 lm halogen tungsten lamps Aluline (Philips, Netherlands) and 10 × 140 lm 567.5 nm (lime color) LUXEON light emitting diodes (Lumileds, Netherlands) was used to illuminate the skin surface. To reduce skin specular surface reflectance, linear polarizers were placed in front of the light source oriented orthogonally to the filter’s polarizer.

Images of the knee area were captured with the camera positioned at a distance of 25–35 cm from the patient’s patella and at a straight angle to the patient’s longitudinal axis. The captured skin area was 50 × 35 mm. 4 × 4 pixel binning was used resulting in 0.05 × 0.05 mm spatial resolution. The skin area imaged was marked by permanent marker as a reference for repeated imaging. All HSI data were saved as a set of lossless monochrome.tiff files.

**Fig. 1:**
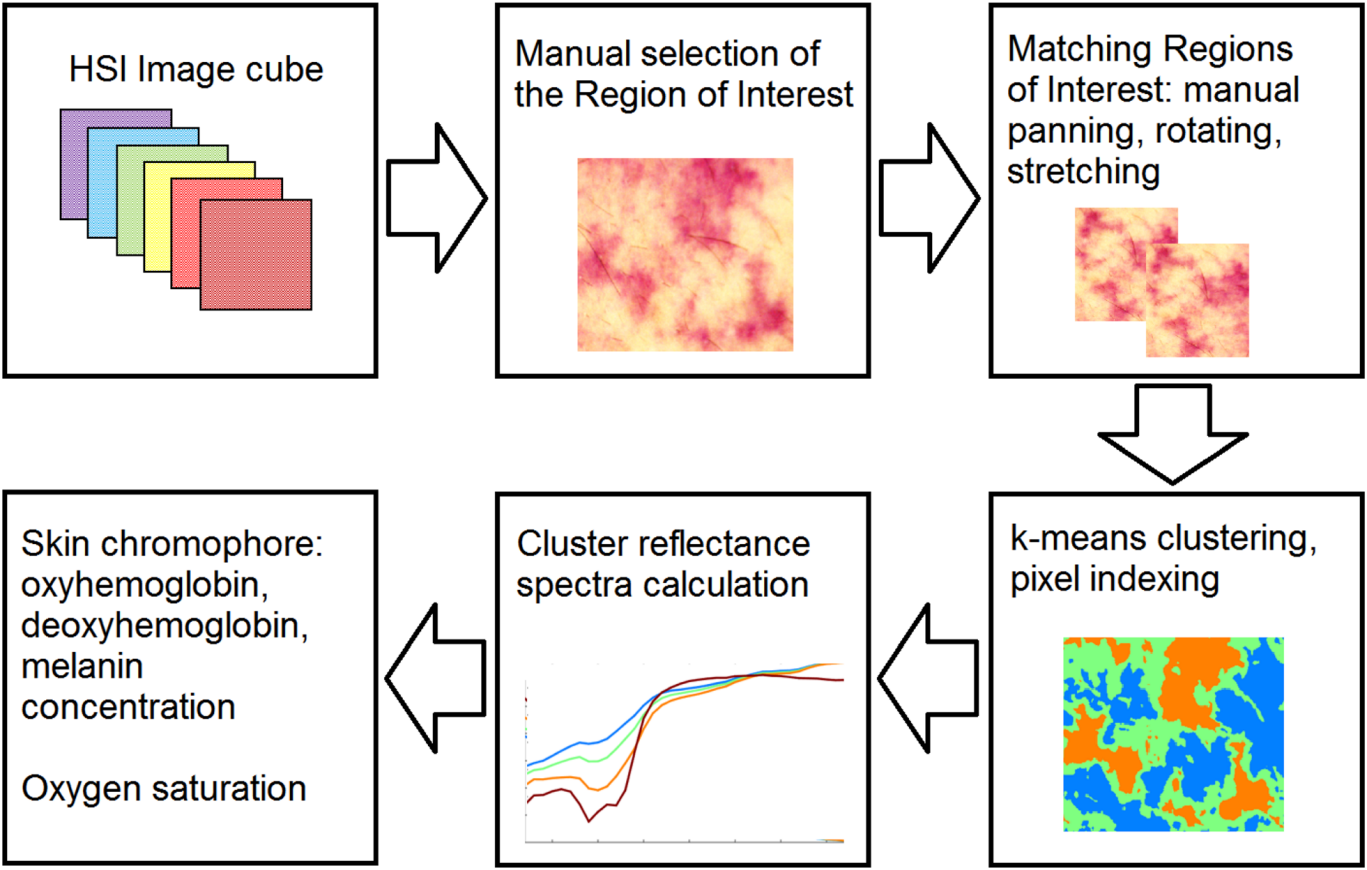
Method of processing of hyperspectral imaging data

The processing of the HSI data was performed offline in semiautomatic mode using custom-developed MATLAB (MathWorks, Natick, MA, USA) code comprising several stages as shown in Fig. 1. Skin in the imaged area was divided into four clusters with similar spectral properties indicating its chromophore content. Regions with low blood circulation (seen as patches of pale skin) were assigned to the first cluster, while skin where blood pooling was typical (skin patches described as blue) was assigned to the fourth cluster. Skin oxygen saturation (μHbO_2_) value was calculated from the fourth cluster with the highest blood content by using the diffusion light transport model based on a three-layer skin structure containing melanin, oxyhemoglobin and deoxyhemoglobin chromophores [16]. This model assumes that light reflection occurs in the papillary dermis and chromophores absorbing light are epidermal melanin and hemoglobins contained in the vessels of the superficial dermal plexus. These vessels are capillary loops of dermal papillae with a diameter of 17–22 μm [17].

### Rationale for study design

The setting of MAP targets in this study was clinician driven. Published data show that clinicians target higher MAP values than recommended by the guidelines [18, 19]. It results in a subgroup of noradrenaline-requiring patients who cannot be defined as being in septic shock as they can maintain MAP of 65 mm Hg without vasopressor support. To be able to include this patient group, we designed the study protocol to target a relative increase in MAP rather than a change in absolute values. We chose to aim for an increase in 20 mm Hg based on previous studies [9, 20].

### Study protocol

Baseline noradrenaline infusion was adjusted to achieve a mean arterial pressure (MAP) of 65 ± 5 mm Hg. In thirteen patients, the lowest achieved baseline value of MAP was > 70 mm Hg when noradrenaline infusion was discontinued during adjustment. Patients with noradrenaline infusion at baseline were included in the septic shock group, patients who had their noradrenaline infusion discontinued into the sepsis group. The resulting set of hemodynamic variables (heart rate, systolic blood pressure, mean arterial pressure, the dose of noradrenaline) was recorded after a 30-min stabilization period and HSI performed. The dose of noradrenaline was then increased by 0.05 mcg/kg/min every 10 min to increase MAP by 20 ± 5 mm Hg. After 30-min stabilization period, a repeated set of hemodynamic variables, dose of noradrenaline and HSI of the same area were obtained.

During adjustment of noradrenaline, patients were monitored for the development of arrhythmias and excessive peripheral vasoconstriction. All other infusions and ventilator settings were maintained unchanged.

All subjects were followed up to 28 days or until death.

### Outcomes

The primary outcome was skin oxygen saturation at baseline MAP and after adjustment of noradrenaline dose to increase MAP by 20 ± 5 mm Hg in relation to disease severity measured by SOFA score. Secondary outcomes were intensive care unit mortality in relation to the effect of increased MAP on μHbO_2_.

### Statistical analysis

Continuous variables are reported as median values and interquartile range (IQR), binary and categorical variables—as counts and proportions. Wilcoxon signed-rank test was used to compare hemodynamic variables and μHbO_2_ at baseline and after study intervention. Kruskal–Wallis and Mann–Whitney U tests were used for comparisons between groups. Multiple linear regression modeling was used to test association between severity of disease measured by SOFA score, intensive care survival and μHbO_2_ at baseline and after adjustment of MAP. *p* < 0.05 was considered statistically significant. Statistical analysis was performed using R version 3.2 (R Foundation for Statistical Computing, Vienna, Austria).

We assumed that skin oxygen saturation and disease severity will be moderately correlated and estimated that a total sample size of ≥ 30 patients would be necessary (*r* = 0.5; *α* = 0.05; 1 − *β* = 0.8).

## Results

We included 30 patients within median 16 h of admission to the intensive care unit and 15 healthy volunteers. Two patients had to be excluded from analysis because of the poor quality of HSI data. Volunteer and patient characteristics by group are shown in Table 1.

**Table 1:** Demographic, clinical and hemodynamic characteristics of study subjects

	All patients (n = 28)	Sepsis (n = 13)	Septic shock (n = 15)	Controls (n = 15)
Demographic and clinical characteristics				
Age (in years)	70 (62–78.5)	70 (65–78)	69 (56–79)	35 (25–36)^†^
Male, *n* (%)	17 (61%)	8 (63%)	7 (47%)	8 (53%)
APACHE II score	23 (18–29)	17 (15–22)*	27 (21–29)	NA
SOFA score	10 (7–12)	7 (6–11)*	11 (10–12)	NA
Arterial lactate, mmol/l	4.0 (2.4–5.3)	5.0 (2.4–7.0)	4.0 (2.3–7.0)	NA
Mechanical ventilation, *n* (%)	7 (25%)	3 (23%)	4 (27%)	NA
Renal replacement therapy, *n* (%)	5 (18%)	2 (15%)	3 (20%)	NA
ICU survivors, *n*, (%)	23 (82%)	11 (85%)	12 (80%)	NA
28-day survivors *n*, (%)	20 (71%)	9 (70%)	11 (65%)	NA
Source of sepsis *n*, (%)				NA
Abdominal	12 (43%)	6 (46%)	6 (40%)	
Respiratory	8 (29%)	3 (23%)	5 (33%)	
Urinary	4 (14%)	3 (23%)	1 (7%)	
Other	4 (14%)	1 (8%)	3 (20%)	
Hemodynamic characteristics				
Baseline systolic blood pressure (mm Hg)	99 (92–107)	100 (98–106)	97 (92–108)	115 (112–127)^†^
Baseline mean arterial pressure (mm Hg)	67 (65–74)	74 (69–79)**	65 (64–67)	91 (87.5–98.5)†
Volume of fluids before inclusion (ml)	2887 (2402–3205)	2402 (2315–2608)	3270 (2428–3420)	NA
Baseline dose of noradrenaline (mcg/kg/min)	0.04 (0–1)	0**	0.1 (0.06–0.25)	NA
Heart rate (beats/min)	113 (92–137)	113 (92–114)	128 (110–145)	63 (59–70.5)^†^
Mottling score				NA
0	22 (79%)	12 (92%)	10 (67%)	
1	2 (7%)	0	2 (13%)	
2	3 (11%)	1 (8%)	2 (13%)	
3	1 (3%)	0	1 (7%)	

Data are presented as numbers with percentages (%) or medians with interquartile range

APACHE II, Acute Physiology, Age, Chronic Health Evaluation II; Sequential Organ Failure Assessment (SOFA) score represents maximum value calculated within 24 h of intensive care unit admission

* *p* < 0.05 sepsis vs. septic shock

** *p* < 0.001 sepsis vs. septic shock

^†^*p* < 0.001 controls vs. sepsis and septic shock

In 16 patients, initial mean arterial pressure exceeded 70 mm Hg and noradrenaline dose had to be reduced before baseline HSI could be performed. In thirteen of these patients, MAP did not drop below 70 mm Hg when noradrenaline was discontinued. The resulting baseline mean arterial pressure of the cohort was 67 (65 to 74) mm Hg. After adjustment of noradrenaline dose according to protocol, mean arterial pressure increased to 85 (83 to 90) mm Hg.

### Response to study intervention

**Fig. 2:**
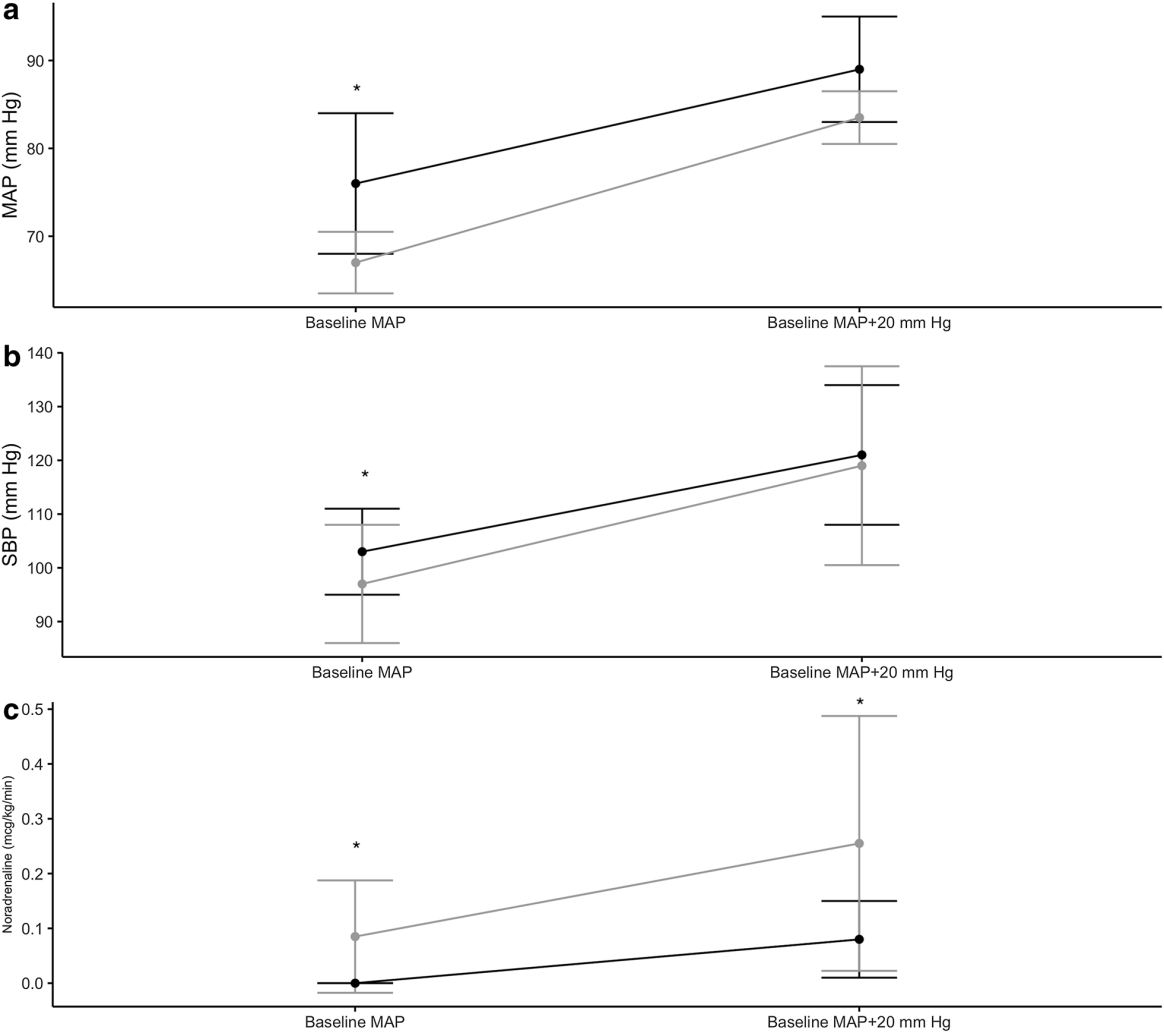
Dynamics of mean arterial pressure (MAP) (**a**), systolic blood pressure (**b**) and dose of noradrenaline (**c**) before and after adjustment of noradrenaline to increase MAP by 20 ± 5 mm Hg in sepsis (black) and septic shock patients (gray). Data are expressed as median and interquartile range. *p < 0.05 sepsis vs. septic shock

Dynamics of systolic arterial pressure, mean arterial pressure and dose of noradrenaline over the course of the study are shown in Fig. 2. Targeting a change in mean arterial pressure of 20 ± 5 mm Hg resulted in a change of systolic arterial pressure from 100 (98–106) to 118 (116–127) mm Hg in the sepsis group and from 97 (92–108) to 122 (113–132) mm Hg in the septic shock group. The median dose of noradrenaline increased from 0 to 0.08 (0.04–0.1) mcg/kg/min and from 0.1 (0.06–0.25) to 0.27 (0.14–0.44) mcg/kg/min, accordingly.

**Fig. 3:**
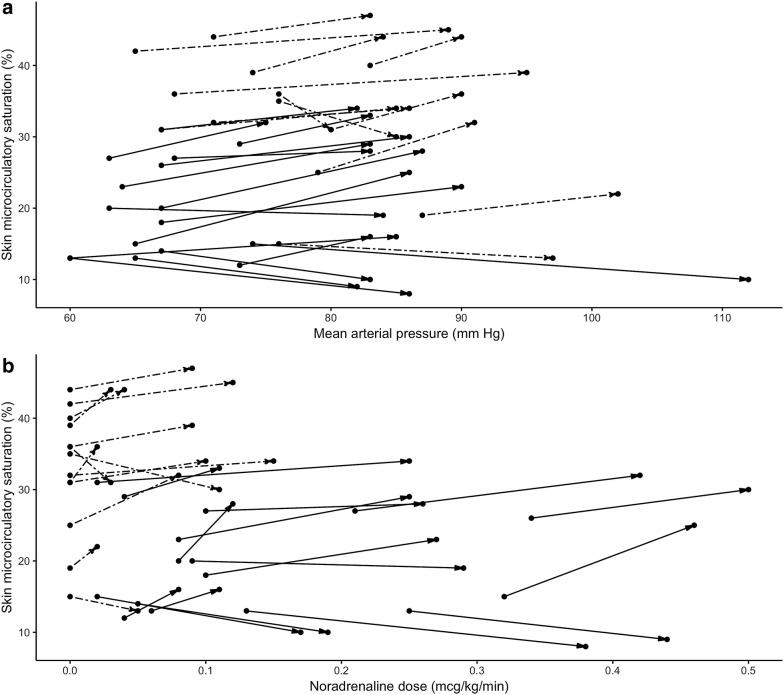
Individual responses of skin oxygen saturation in sepsis (solid lines) and septic shock patients (dashed lines) to changes in **a** mean arterial pressure, and **b** dose of noradrenaline

Baseline skin μHbO_2_ in the patient cohort ranged from 12 to 44%. It was significantly lower in sepsis and septic shock groups, compared to healthy volunteers (35.0 (31.0–39.0) %, 18.0 (13.0–26.0) % and 85.0 (81.0–89.0) %; *p* < 0.001). Improvement of skin μHbO_2_ after study intervention was seen in 21 of 28 patients, 10 in the sepsis group and 11 in the septic shock group. Overall, skin μHbO_2_ changed from 26.0 (24.5–27.0) % at baseline to 30.0 (29.0–31.0) % after noradrenaline dose adjustment (*p* = 0.04). Change in skin μHbO_2_ did not differ between the sepsis and septic shock groups (*p* = 0.69). Changes of μHbO_2_ in individual patients depending on changes in mean arterial, systolic pressure and dose of noradrenaline are shown in Fig. 3.

### Skin microcirculatory response, severity of disease and outcome

**Fig. 4:**
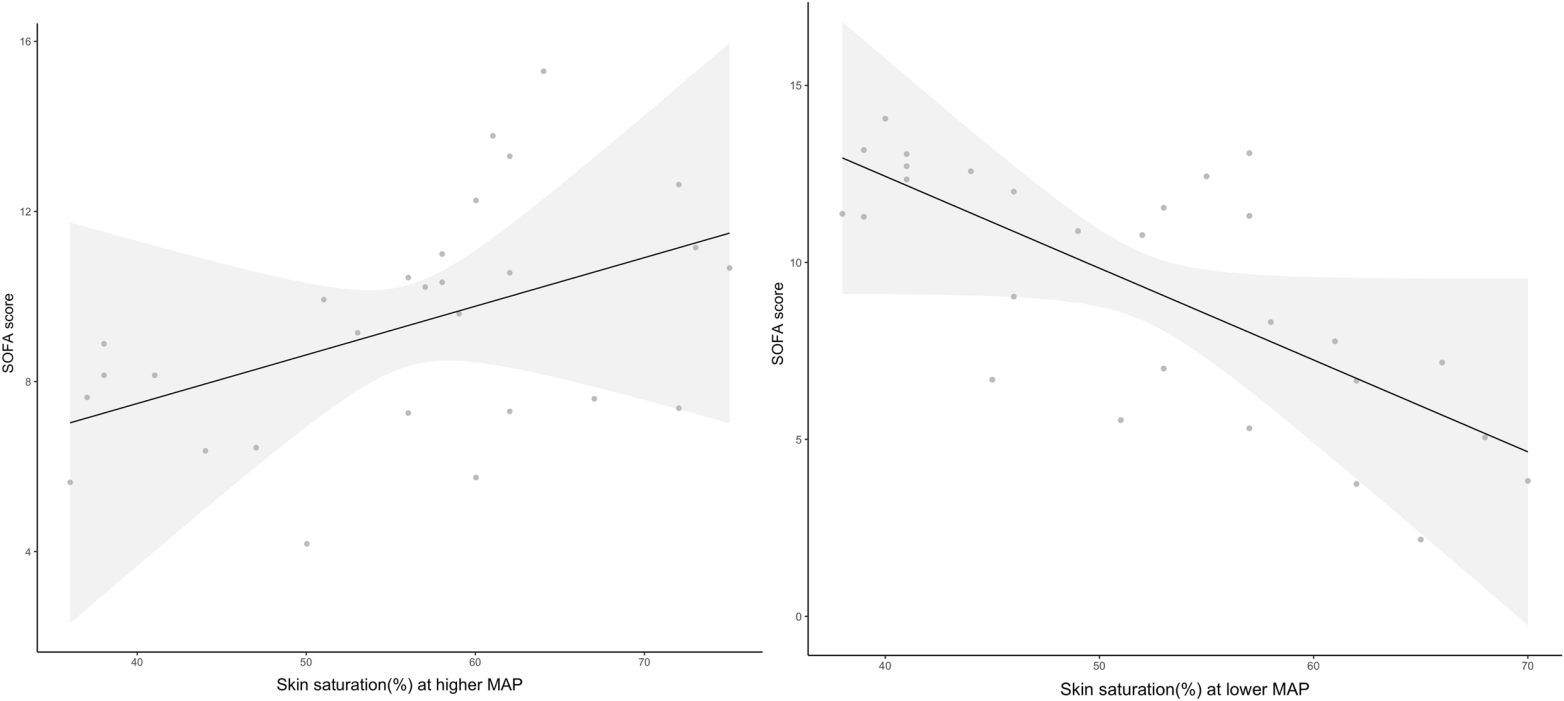
Relationships between skin oxygen saturation at lower (baseline) and higher (baseline + 20 mm Hg) arterial pressure modeled by multiple linear regression. Gray area represents 95th percentile confidence boundaries

Figure 4 shows the relationship between skin μHbO_2_ at lower and higher MAP and disease severity measured by SOFA score modeled by multiple linear regression. Lower baseline skin μHbO_2_ was significantly associated with higher severity of illness as measured by SOFA score. When MAP was increased by 20 mm Hg and adjustment made for baseline μHbO_2_, patients with higher SOFA scores had higher skin μHbO_2_ (*x* = 12.36 − 0.23 × *x*_1_ + 0.1 × *x*_2_; *r*^2^ = 0.21; *p* = 0.02).

Skin μHbO_2_ measured at higher MAP was found to be marginally predictive of mortality (OR: 1.10; 95% CI 1.00–1.23; *p* = 0.053).

## Discussion

The objective of this study was to evaluate the effects of increasing mean arterial pressure by titration of noradrenaline dose on microcirculatory oxygenation of the skin in vasopressor-requiring patients with sepsis using hyperspectral imaging.

In our study septic patients, all of whom had achieved macro hemodynamic resuscitation targets, had significantly lower skin μHbO_2_ compared to healthy controls. As microcirculatory oxygenation is the reflection of tissue oxygen supply and demand, we hypothesize that lower values reflect suboptimal oxygen supply at least in some patients and increase their risk of organ failure. Indeed, in this study higher severity of organ failure measured by SOFA score was associated with lower skin oxygen saturation values. Similar to our study, low thenar muscle oxygen saturation measured by near-infrared spectroscopy (NIRS) was found to be present in 60% of patients with sepsis [21], but only dynamic changes in tissue oxygenation after vascular occlusion test have been shown to be related to organ failure [22]. In our study, we targeted measurement only to the most hypoxic areas of the skin, and our data support a more direct link between hypoxia in microcirculatory beds and development of multiple organ failure.

Several techniques can be used to assess skin microcirculation in critically ill patients. Comprehensive assessment of microcirculation should include the magnitude of microcirculatory flow and oxygenation, functional capillary density and extent of heterogeneity [23]. Laser Doppler and laser speckle imaging allow to quantify microvascular flow, although absolute values of flow cannot be obtained. The advantage of these techniques lies in the ability to quantify relative changes of flow, as can occur during the dynamic response to a noradrenaline challenge [24]. Yet, the goal of microvascular resuscitation is to recruit non-perfused vessels rather than increase flow in already perfused ones. In this respect, the optical monitoring of microcirculatory oxygenation might be more informative. NIRS can be used to measure muscle oxygenation. However, NIRS measures a composite value of saturation in a volume of tissue under the probe [25]. With this technique, oxygenation can be overestimated in cases of heterogeneity, as can be present in septic shock. The presence of skin mottling around the knee is an easy way to assess heterogeneity of skin oxygen saturation and pooling of blood. Mottling score allows semiquantitative evaluation of the extent of mottling with higher values shown to be associated with 14-day mortality in patients with severe sepsis [26]. The main limitation of clinical assessment of mottling is the inability to make quantitative measurements. Hyperspectral imaging of the skin overcomes limitations of clinical assessment and allows to map and perform targeted measurements of skin microvascular oxygenation. Our study shows that HSI technology can be used to detect individual changes in μHbO_2_ in response to change in perfusion pressure.

In sepsis achievement of adequate systemic perfusion pressure is considered crucial during the early phase of hemodynamic resuscitation. The newest update of Surviving Sepsis Campaign guidelines recommends early introduction of vasopressors to meet the mean arterial pressure target of at least 65 mm Hg [27]. Nonetheless, clinicians frequently choose to increase MAP beyond the recommended limit. Data from several sepsis trials show that average values of above 75 mm Hg were achieved, even though the targeted range was 65 to 70 mm Hg [18, 19]. Hemodynamic management of patients before enrollment into our study was clinician driven, and in 13 patients noradrenaline was administered to target MAP > 70 mm Hg. This subgroup could maintain baseline MAP of 65 mm Hg without vasopressor support. However, increase in MAP by 20 mm Hg resulted in improvement of skin oxygen saturation in 21 of 28 patients, by 2% in the overall, and there were non-responders in both sepsis and septic shock groups. Similar improvements of microcirculatory perfusion and oxygenation with noradrenaline challenge have been described with much higher MAP early in the course of sepsis. Jhanji et al. reported an increase in cutaneous tissue oxygen pressure from 44 to 54 mm Hg in septic shock patients when targeting mean arterial pressures of 60 and 80 mm Hg, accordingly [10]. Thooft et al. studied the effects of changes in arterial pressure and found no overall effect on thenar muscle saturation but response varied between patients [9]. After the increase in MAP with adjustment for baseline μHbO_2_ in our study, more improvement in oxygen saturation values was achieved by patients with higher SOFA scores. The study was not powered to detect differences in mortality; yet, lower skin oxygenation at MAP of 85 mm Hg was associated with higher mortality. These findings suggest that the most severely ill patients might benefit more from manipulation of blood pressure to increase their tissue oxygen supply. This effect seems to be variable, and low μHbO_2_ values even at high MAP convey a poor prognosis.

A small proportion of patients had a reduction in skin oxygenation with doses of noradrenaline required to increase MAP to 85 mm Hg. Such variability of response has been shown in previous research [9] and most likely depends on the basal state of microcirculation. Findings of variability from our and other studies suggest that monitoring of microcirculation might be especially important if high vasopressor doses are used or higher MAP values targeted. Any benefits of increasing perfusion pressure by using catecholamines should be weighed against possible deleterious effects of excessive vasoconstriction. Monitoring of microcirculation is essential to achieve a balance between improved peripheral perfusion and harmful effects of excessive sympathetic stimulation.

The values of skin μHbO_2_ should be interpreted with caution as they are significantly lower than those reported by other technologies, such as NIRS. A meta-analysis of near-infrared spectroscopy in septic patients by Neto et al. reported baseline value of thenar oxygen saturation of 78% in patients with septic shock [28]. Previous data regarding absolute values of skin oxygenation measured by HSI are limited. The control group used in this study had lower extremity skin μHbO_2_ values of 81 to 89% which are significantly higher than 40 to 50% in a previous report [25]. Information regarding skin μHbSO_2_ measured by HSI in critical illness is available from porcine model of hemorrhagic shock. Cancio et al. showed a linear decrease of μHbO_2_ with progressive blood withdrawal; the minimum value observed was 19% [29]. Skin μHbO_2_ values observed in our study seem to fall within the range described in the animal model of shock.

There are several limitations to this study. A subgroup of patients who could maintain baseline MAP of 65 mm Hg without vasopressor support was included. That resulted in significant differences between patients in terms of initial hemodynamic status. In comparison with other studies investigating the effects of increasing MAP on microcirculation, our study did not use predefined MAP values but aimed to increase MAP by 20 mm Hg from baseline. Nevertheless, like studies which included only patients with septic shock and used predefined MAP values, we obtained a significant response in μHbO_2_ after increase in MAP and these changes were not limited to patients with more severe cardiovascular failure. The study cohort was also small but comparable to other similar studies [9, 10]. This is a reflection of the number of potentially recruitable patients in a single intensive care unit study. The design of the study did not involve evaluation whether effects of changes in skin μHbO_2_ were associated with longer-term changes in organ dysfunction. Whether short-term increase in skin microcirculatory oxygenation leads to improved outcomes has yet to be shown.

The potential of hyperspectral imaging to quantitatively evaluate skin perfusion and oxygenation in critically ill has not been fully explored. Yet, results from our study need to be replicated in a larger and more diverse patient population with determination of critical cutoff values and confirmation that targeting microcirculatory oxygenation in early sepsis results in better clinical outcomes in patients with septic shock.

## Conclusions

We have shown improvement of skin microcirculatory oxygenation measured by hyperspectral imaging with increase in mean arterial pressure in a cohort of patients with sepsis. Non-survivors had lower skin μHbO_2_ values at baseline, but patients with more severe sepsis-related organ damage showed greater improvement in μHbO_2_ when mean arterial pressure was increased by 20 mm Hg.

## Data Availability

All the data supporting our findings are available from corresponding author upon reasonable request.
